# Traditional Mongolian swaddling and developmental dysplasia of the hip: a randomized controlled trial

**DOI:** 10.1186/s12887-021-02910-x

**Published:** 2021-10-13

**Authors:** Munkhtulga Ulziibat, Bayalag Munkhuu, Ariun-Erdene Bataa, Raoul Schmid, Thomas Baumann, Stefan Essig

**Affiliations:** 1 Department of Health Sciences and Medicine, University of Lucerne, Luzern, Switzerland; 2 National Center for Maternal and Child Health, Ulaanbaatar, Mongolia; 3 Baarer Kinderarztpraxis, Baar, Switzerland; 4 St. Niklausstr. 12 4500, Solothurn, Switzerland; 5 Center for Primary and Community Care, Department of Health Sciences and Medicine, University of Lucerne, Luzern, Switzerland

**Keywords:** Developmental dysplasia of the hip, Swaddling, Hip ultrasound examination

## Abstract

**Background:**

Mongolian traditional swaddling of infants, where arms and legs are extended with a tight wrapping and hips are in adduction position, may lead to abnormal maturation and formation of the hip joint; and is a contributing factor for developmental dysplasia of the hip (DDH). This hypothesis was tested in this randomized controlled trial.

**Methods:**

Eighty newborns with one or two hips at risk of worsening to DDH (Graf Type 2a; physiologically immature hips) at birth were randomized into 2 groups at a tertiary hospital in Ulaanbaatar. The “swaddling” group (*n* = 40) was swaddled in the common traditional Mongolian method for a month while the “non-swaddling” group (*n* = 40) was instructed not to swaddle at all. All enrollees were followed up on monthly basis by hip ultrasound and treated with an abduction-flexion splint if necessary. The groups were compared on the rate of Graf’s “non-Type 1” hips at follow-up controls as the primary outcome. Secondary outcomes were rate of DDH and time to discharge (Graf Type 1; healthy hips). In addition, correlation between the primary outcome and swaddling length in days and frequency of swaddling in hours per day were calculated.

**Results:**

Recruitment continued from September 2019 to March 2020 and follow-up data were completed in June 2020. We collected final outcome data in all 80 enrollees. Percentages of cases with non-Type 1 hip at any follow-up examination were 7.5% (3/40) in the non-swaddling group and 40% (16/40) in the swaddling group (*p* = 0.001). There was no DDH case in the non-swaddling group while there were 8 cases of DDH in the swaddling group. The mean time to discharge was 5.1 ± 0.3 weeks in the non-swaddling group and 8.4 ± 0.89 weeks in the swaddling group (*p* = 0.001). There is a correlation between the primary outcome and the swaddling frequency in hours per day (r = 0.81) and swaddling length in days (r = 0.43).

**Conclusions:**

Mongolian traditional swaddling where legs are extended and hips are in extension and adduction position increases the risk for DDH.

**Trial registration:**

Retrospectively registered, ISRCTN11228572.

## Background

Developmental dysplasia of the hip (DDH) belongs to the most common disorders of the osteoarticular system with public health priorities in otherwise healthy infants. Severity can range from a minor acetabular dysplasia to a complete dislocation. Estimates of the incidence of DDH are quite variable and depend on detection methods, ages of the child, and diagnostic criteria [1, 2]. Mongolian studies reported 1.2 to 1.3% incidence of DDH (Type 2c, D, 3 and 4) by Graf method of ultrasound which is comparable to that in European neonates (1–2%) [3, 4]. DDH is a multifactorial disorder with genetic and non-genetic factors that are involved in its etiology [5]. Contributing factors for the development of DDH are limited space (big infants and breech delivery) and hormonal factors (Relaxin) [6].

**Fig. 1:**
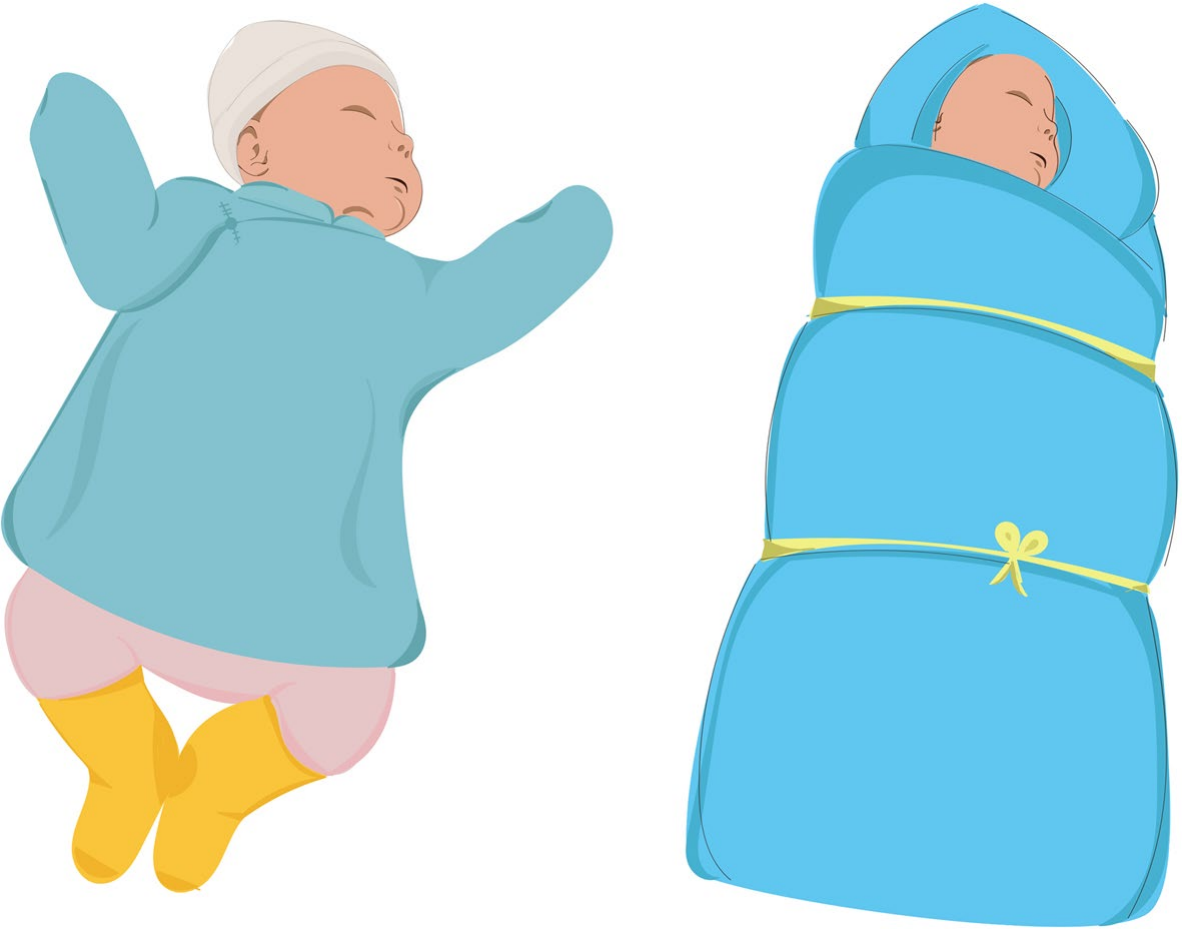
Non-swaddled baby and Mongolian traditional swaddled baby

In many cultures around the world newborn babies are swaddled to calm them down and fall asleep, and some cultures believe that swaddling can prevent the baby from suffering from cold weather especially during winter [7]. In Mongolia, swaddling is an ancient practice and nowadays still remains common child care in the first months of life, especially among rural nomadic families. The traditional way of swaddling technique involves tight, prolonged wrapping from the head or neck down in two to three layers of thin cotton cloth, covered by layers of thick blankets and binding with 2–3 cords (Fig. 1).

However, improper swaddling may increase the risk of hip dysplasia and hip dislocation [8, 9]. A systematic literature review regarding DDH indicates that continued use of improper swaddling techniques, such as straight-legged swaddling, is a significant risk factor for the development of DDH in observational studies [2]. A recent study in Mongolia showed that around 15% of screened 230,079 newborns have physiologically immature hips (Graf’s Type 2a hips) at birth. Among the physiological immaturity approximately 1–2% degrade and develop DDH and need treatment with a flexion-abduction orthosis [4]. In these cases, unfavorable mechanical conditions may exist after birth since Mongolian traditional swaddling requires a tight wrapping with extended legs and the hips are held in an adducted position. Therefore, traditional swaddling possibly increases the risk for DDH. This hypothesis was tested in this randomized controlled trial.

## Methods

### Study design

Null hypothesis: Mongolian traditional prolonged swaddling where arms and legs are extended and hips are in adduction position does not increase the risk for DDH.

Prospective interventional randomized controlled trial with two study groups. The study protocol was reviewed and approved by the Institutional Review Board at the National Center for Maternal and Child Health (04/2019) and the Ethical Review Committee of Ministry of Health, Mongolia (133/2019). Registration number: ISRCTN11228572.

### Study site

The National Center for Maternal and Child Health (NCMCH) is purposely selected as a study site. Approximately 35% of all deliveries in Ulaanbaatar, the capital city, occur at the hospital and there are about 12,000 live births annually [10]. As the hospital is a specialized tertiary care level teaching hospital, it is responsible for the nationwide surveillance of newborn hip ultrasound screening, follow-up ultrasound controls and management of DDH cases.

### Study participants

#### Sample size calculations

The primary outcome of interest is dichotomous: Graf Type 1 or non-Type 1 hips during follow-up. If we assume that 10% of the subjects of the non-swaddled group develop a non-Type 1 hip and it is of clinical relevance only if we observe an effect size of 30% difference (i.e. 40% of the swaddled group will develop a non-type 1 hip), 58 participants were needed to reach 80% power at a significance level of 5%. To increase the statistical power, and assuming 20% dropout, it was decided to include about 80 (40 in each group) subjects.

#### Examination and randomization

**Fig. 2:**
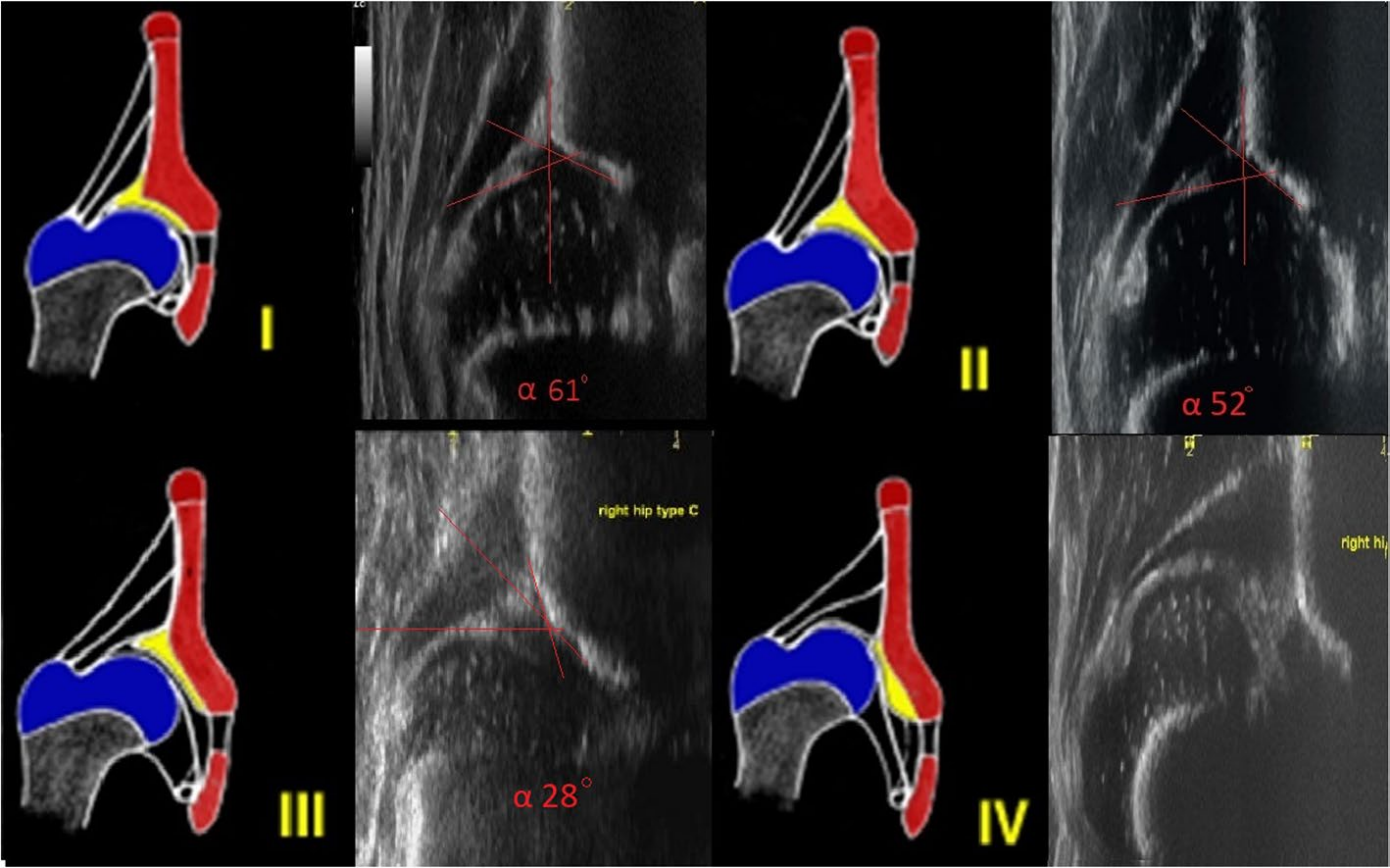
Graf’s staging

There are 6 neonatologists/screeners at post-delivery wards of the hospital who perform the hip ultrasound screening. Out of the 6 neonatologists/screeners, only one neonatologist was assigned as a data collector for the study to reduce a baseline diagnostic error. On the first days after birth, all newborns of the data collector were screened using Graf’s method of ultrasound according to the Mongolian national guidelines [4]. The standardized and uniform system of Graf (Fig. 2) was used for diagnostics [11].

After the screening, we generated a list of all newborns with Graf Type 2a hips on daily basis. All term newborns with Graf Type 2a (physiologically immature) hips were eligible for the study. Predefined exclusion criteria were: parents known to be unable to return for the scheduled follow-up ultrasound examination during the study period, unwilling to give informed consent, newborns with obvious congenital abnormalities (with clear medical consequences), newborns with needs for intensive care treatment, and newborns with low birth-weight (≤2500 g). A randomization sequence was created using Excel 2007 (Microsoft, Redmond, WA, USA) with a 1:1 allocation using random block sizes of 4 by an independent researcher with no clinical involvement in the trial. After obtaining the consent from the parents, details of the allocated group were given on colored cards contained in sequentially numbered and sealed envelopes. These were prepared by the principal investigator and kept in an agreed location on the post-delivery ward. Randomization took place before discharge when the data collector/neonatologist gives detailed consultation on usual postnatal care of newborns. Corresponding envelopes were opened only after the enrolled newborns completed all baseline assessments and it was time to allocate the intervention. Parents allocated to the swaddling group and the research assistants were aware of the allocated arm. The outcome assessor/radiologist were kept blinded to the allocation.

#### Swaddling group

The newborns randomized into the “swaddling” group were instructed to swaddle in the common traditional Mongolian technique (Fig. 1) at least 20 h every day for around a month (it was not allowed to continue the swaddling more than 1 month for the study purpose because of the Ethical Review Committee’s recommendations). The swaddled group received a blanket and several cotton sheets at the time of recruitment.

#### Non-swaddling group

The “non-swaddling” group were instructed not to swaddle at all for around a month. The required size and warmth of clothing required to keep a newborn infant warm were provided to the family. Also, wide warm sleeping bags that allow free legs movements were provided to prevent swaddling when going outdoors.

### Quality control and data

The hip ultrasound examination was performed by a trained and experienced neonatologist using an ultrasound machine (GE Logiq series) with a linear array transducer operating on an ultrasound frequency of 7–10 MHz. A Sonofirst holding cradle and transducer fixation unit (Orthopunkt, Solothurn) was used to prevent tilting errors and to standardize examination techniques. A web-based, password-protected platform, “HipScreen” (WebWaren, Bern, Switzerland) [12] was used for documentation and quality control purpose. The tool enabled the screener to upload DICOM files exported from the ultrasound machines. Four images (two per hip side; one of them with measured alpha and beta angles according to Graf), were required. This allowed continuous and reliable review of hip grading, diagnosis of DDH, and treatment decisions. Trained, on-site experts checked all examinations on the platform and promptly sent comments to the screener. Discrepancies between assessments were resolved by discussion and consensus.

Age at hip ultrasound screening in days, sex, possible risk factors for DDH (birth weight ≥ 4000 g, breech delivery, family history of DDH, first born) and hip data (alpha angles at baseline and management) of all newborns were recorded at baseline. The newborns and their mothers underwent a screening process by research assistants to see whether they meet the inclusion criteria. The parents were given oral information about hip dysplasia and study purpose. The parents were requested to sign an informed consent form if they agree to take part in the study. Those who refused consent were registered but not included. After the randomization, the research assistants explained and gave detailed information about the procedures according to both “swaddled” and “non-swaddled” groups. For babies assigned to the “swaddling” group, parents were instructed to follow a pattern of traditional Mongolian swaddling as described above. For babies assigned to the “non-swaddling” group, parents were instructed to do not swaddle at all and only use a wide sleeping bag with free leg position.

It was expected that some families would not adhere to their instructions of swaddling or not swaddling, mainly because of the pressure of grandparents and other cultural factors. To monitor compliance and obtain a measure of exposure, multiple sources of information were used. First, mothers prospectively filled out a 7-days diary for swaddling/clothing which was collected by research assistants during weekly home visits. Second, a retrospective 24-h history of swaddling/clothing based on the parent’s description was collected. For the analysis, the exposure recordings were combined to reveal exposure to swaddling/clothing per child.

### Outcome assessment

The primary outcome of the study was the number of children with “non-Type 1” hips at follow-up visits. At 4–7 weeks, all babies were checked by Graf’s method of hip ultrasound performed by an experienced sonographer uninformed about the initial status of the hips. Regardless of the study group, monthly controls were fixed until complete maturation to Graf type 1 was documented. All infants who developed DDH (defined as infants whose Graf’s ultrasound type is Type 2a-, 2c, D, 3, or 4) were treated with a flexion-abduction splint (Tübingen, a hygienic and washable, reusable hip flexion-abduction orthosis). Families, who did not show up for follow-up were contacted by phone or visited at home. In 21 cases when the parents were not able/willing to come to the 1st follow-up control visit, the PI visited the family and made the follow-up ultrasound examination at their home using a portable ultrasound device (MicroUs EXT-1H, REV:C, Vilnius Lithuania).

Secondary outcomes were the number of children with DDH at any monthly follow-up ultrasound control visits, and time to healthy hips (time between enrolment and discharge with Type 1 hips). In addition, a correlation between the primary outcome and length of swaddling in days and a correlation between the primary outcome and swaddling frequency in hours per day were calculated in the swaddling group.

### Operational definitions

*Graf Type 2a hip* (physiologically immature): has a certain degree of physiological delay in ossification of the bony acetabular roof. The alpha angle is between 50 and 59, the beta angle is between 55 and 77 degrees, and the patient is younger than 6 weeks of age [11].

*Graf Types 2a- and 2a+:* The differentiation of Type 2a + and Type 2a- is made after the 6th week. Starting from 55° alpha at 6 weeks of age and assuming a spontaneous maturation of 1° per week. Type 2a + is still considered to be physiological while 2a- hips are assigned to the group of DDH [11].

### Statistical analysis

Data were double entered using EpiData (The EpiData Association Odense, Denmark) and analyzed using Stata 16 (Stata Corporation). Balance checks for the “swaddling” and “non-swaddling” groups were conducted and reported for all variables measured at baseline. Outcomes were evaluated in an intention-to-treat analysis. Data are expressed as means with standard deviations for data with normal distributions and as medians with interquartile ranges (IQR) for non-normal distributions. For continuous variables, groups were compared using t-tests. For categorical variables, the χ^2^ test and Fisher’s exact test were used.

For the primary outcome, the risk of “non-Type 1” was determined by the Binary logistic regression analysis adjusted for the mentioned baseline variables. For the secondary outcomes, a hazard ratio was calculated using a Cox proportional hazard model to assess the risk associated with DDH. Kaplan-Meier analysis estimated the cumulative incidence of time to healthy hips in weeks; survival curves were calculated and compared using a log-rank (Mantel-Cox) test. For the additional analyses, Pearson’s correlation coefficients were calculated between the primary outcome and swaddling frequency in days and swaddling length per day in hours.

All probability values were considered significant for *p* < 0.05. We used the child (not the hip) as the unit of analysis. If a child had hips with different morphologies, we evaluated that child based on the worse hip.

## Results

**Fig. 3:**
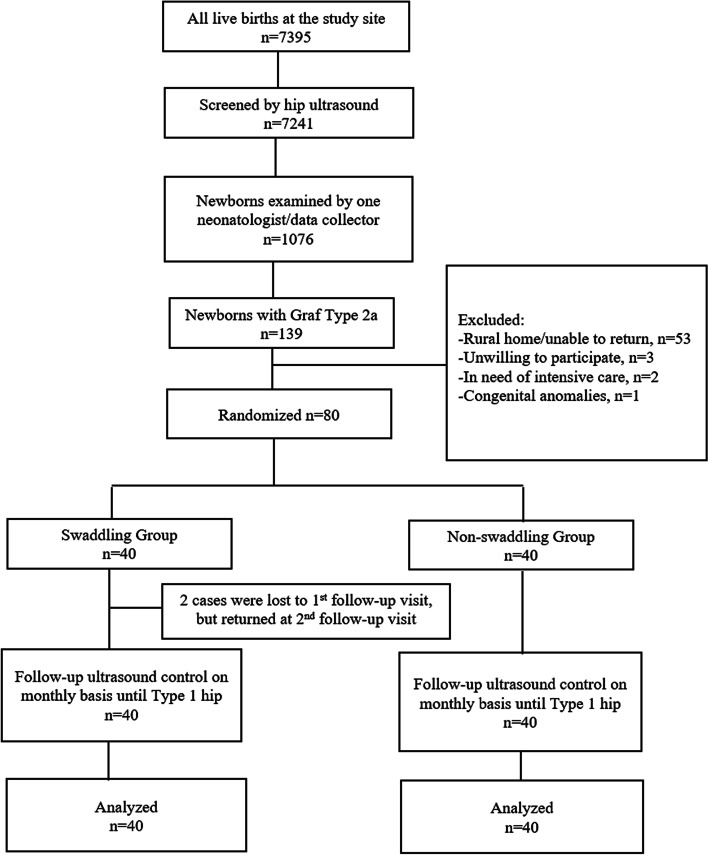
Trial profile

Recruitment started in September 2019 until March 2020 and all follow-ups were completed in June 2020. Figure 3 shows the flow of study clusters and eligible infants throughout the trial. Two cases of the swaddling group who were lost at first follow up visit were brought back at the second follow-up control. The reason of the lost to first follow-up was the families’ moving to rural provinces. Since the two newborns were brought back after 2 months, we could analyze outcome data in all enrollees (Fig. 3).

### Baseline characteristics

The mean age of the recruited 80 newborns was 1.3 ± 0.6 days (range, 1–4 days) and there was no significant difference in mean age between the non-swaddling (1.1 ± 0.14 days) and swaddling (1.0 ± 0.13 day) groups. Among the 80 newborns 42 had unilateral physiologically immature hips while 38 had bilateral physiologically immature hips at birth. The study groups were well balanced for distribution of baseline characteristics at initial screening (Table 1).

**Table 1:** Baseline characteristics of participants by study groups

Characteristics	Non-swaddling N = 40 n(%)	Swaddling N = 40 n(%)	***P***
Mean age in days at screening (mean ± SD)	1.1 ± 0.14	1.0 ± 0.13	0.53
Girls^a^	32(80.0)	34(85.0)	0.56
Breech position^b^	1(2.5)	1(2.5)	0.75
Family history of DDH^b^	1(2.5)	3(7.5)	0.31
Birth weight > 4000gr^b^	5(12.5)	4(10.0)	0.72
Firstborn baby^a^	9(22.5)	4(10.0)	0.13
Mean of Graf’s hip alpha angle at initial screening (mean ± SD)^c^			
Right hip	57.8 ± 4.1	56.7 ± 3.4	0.18
Left hip	56.6 ± 3.8	55.4 ± 3.9	0.19

^a^ Chi-square test, ^b^ Fishier exact test, ^c^ t test; *SD* Standard deviation, *DDH* Developmental Dysplasia of the Hip

### Descriptive development of hip types

**Fig. 4:**
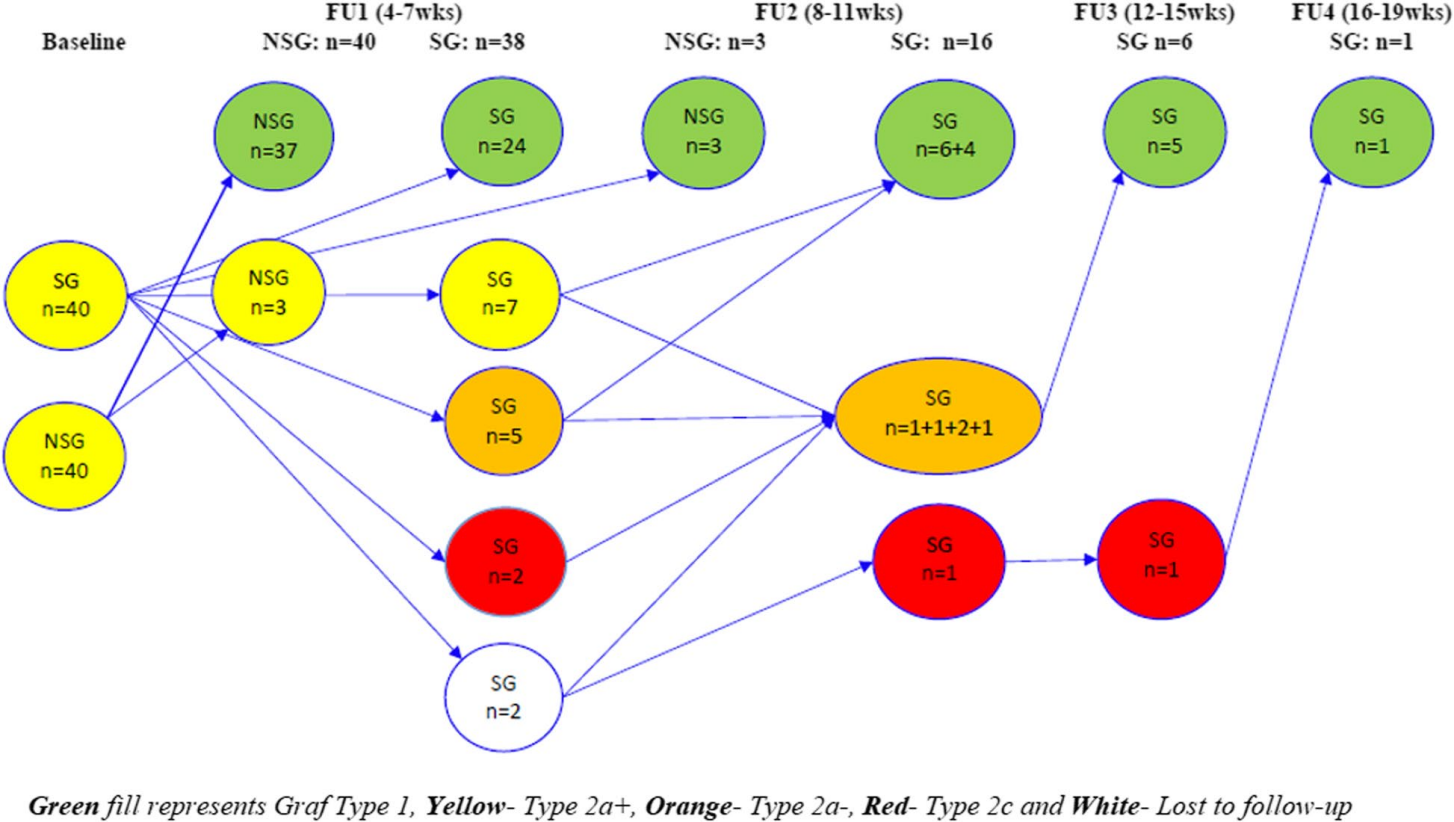
Descriptive development of hips in intervention and control group

The mean age at first follow-up (hip ultrasound control; FU1) of “non-swaddling” and “swaddling” groups was 4.6 ± 0.6 weeks and 4.8 ± 0.9 weeks (*p* = 0.28), respectively. As shown in Fig. 4, of 40 babies recruited in each group, the proportion of testing at FU1 was 100% (40/40) in the non-swaddling and 95% (38/40) in the swaddling group. Among the 40 non-swaddled babies, 37 had developed Type 1 hips; and 3 still had Type 2a hips. Among the 40 swaddled babies, 24 had developed Type 1 hips; 7 still had Type 2a hips; 5 cases were diagnosed with Type 2a-; 2 had Type 2c hips and 2 cases were lost to follow-up. All 61 newborns with Type 1 hips were discharged without further need of treatment or follow-up care in both groups. Ten children (3 in non-swaddling and 7 in swaddling group) with Type 2a + hips continued to be followed up with monthly ultrasound, while 5 children with Type 2a- and 2 children Type 2c (all in swaddling group) were treated with a Tübingen hip flexion splint and followed up with monthly ultrasound. After FU1, all parents stopped swaddling according to the ethical committee (as described in the methods section).

The mean age at second follow-up (FU2) was 10.1 ± 0.14 weeks in the non-swaddling group and 9.4 ± 0.4 weeks in the swaddling group (*p* = 0.06). At FU2, all 3 Type 2a + hips of the non-swaddling group had matured to Type 1 hips. Also 6 out of 7 Type 2a + hips, and 4 out of 5 Type 2a- hips of the swaddling group developed to Type 1 hips. The other 4 hips developed to Type 2a- hips and were treated with Tübingen splint. The 2 cases lost to follow up were brought back to the second follow-up. One was diagnosed with Type 2a-, the other with Type 2c hips. Interestingly, both children were still swaddled until FU2. In those cases, we started treatment with a Tübingen hip flexion splint. All 13 children with bilateral Type 1 hips were discharged. All Type 2a- and Type 2c hips were again treated with a Tübingen hip flexion splint and followed up with monthly ultrasound.

At the third follow up (FU3), the hips of all intervention group children were healed with Type 1 hips and discharged except the one case with Type 2c hips lost to FU1. Consequently, the child also had mature hips after another month treatment with the Tübingen splint at the fourth follow up (FU4).

### Primary outcome

The primary outcome of the study was the number of children with non-Type 1 hip. The percentages of the infants whose Graf’s ultrasound type is other than Type 1 at any monthly follow-up ultrasound control visits were 7.5% (95% CI 1.5–20.4%) in the non-swaddling group and 40% (95% CI 24.9–56.7%) in the swaddling group (*p* = 0.001). The adjusted odds of “non-Type 1” at any monthly follow-up was significantly increased in the swaddling group (OR = 8.65, 95% CI 2.23–33.57; *p* = 0.002).

### Secondary outcomes

**Fig. 5:**
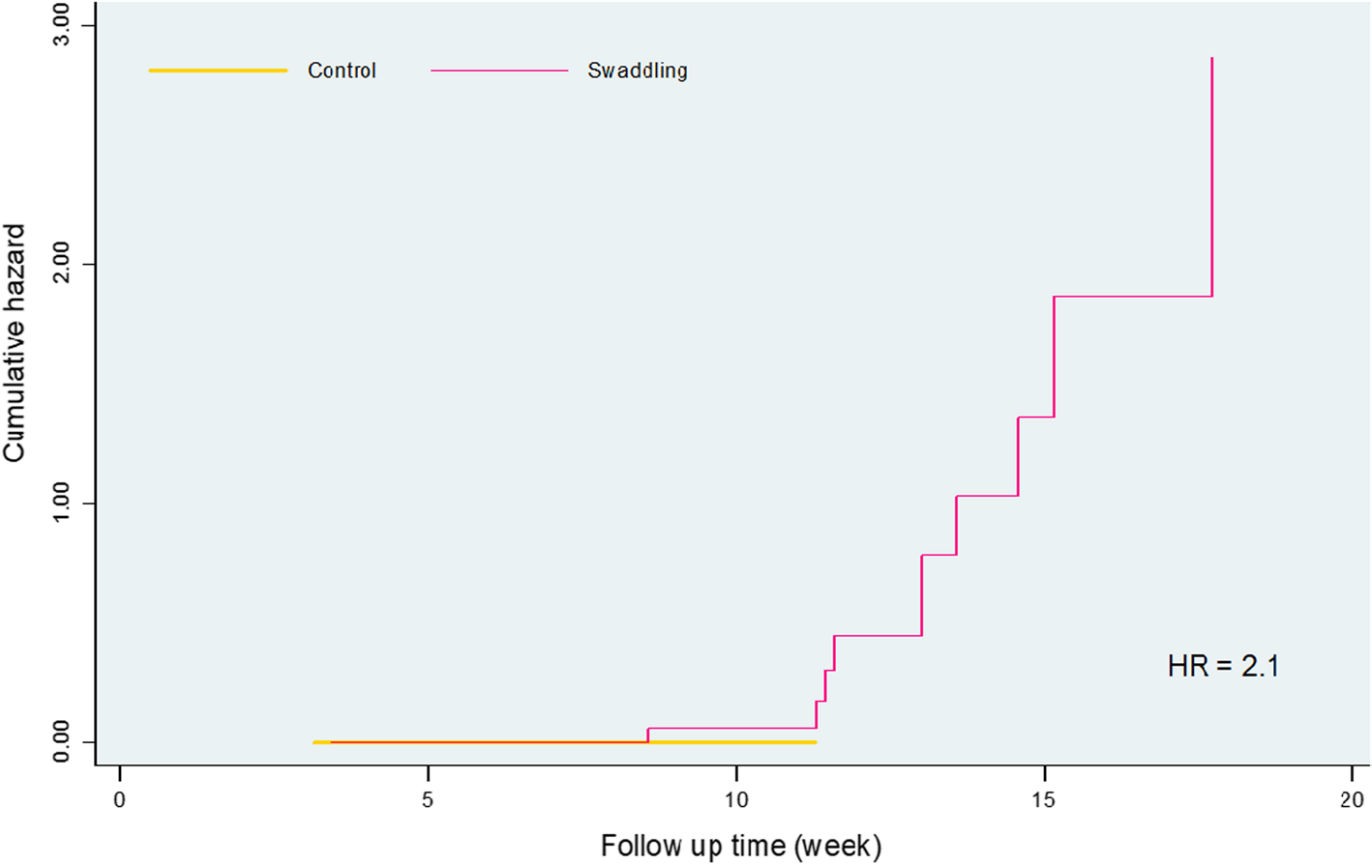
DDH cumulative hazard of the study groups estimated using the Cox proportional hazards regression model

There was no DDH case in the non-swaddling group while there were 8 (20, 95% CI 9.0–35.6%) cases of DDH in the swaddling group. The prolonged tight swaddling group showed a twofold higher hazard risk of DDH compared with non-swaddling group (Fig. 5).

The mean time to Type 1 hips (discharge with healthy hips) was 5.1 ± 0.3 weeks in the non-swaddling group and 8.4 ± 0.89 weeks in the swaddling group (*p* = 0.001; Table 2). The non-swaddling provides 55.8% reduction of the time needed compared to the swaddling group (Hazard Ratio; 95% CI: 0.42; 0.24–0.72, p = 0.001).

**Table 2:** Mean and median of time to Type 1 hips (discharge) in weeks by study groups

Variables	Means	Means and Medians for time to Type 1 hips (discharge)							***P***^**а**^
		Std. Error	95% CI		Median	Std. Error	95% CI		
			Lower	Upper			Lower	Upper	
Control	5.08	0.29	4.50	5.67	4.43	0.06	4.32	4.54	0.001
Swaddling	8.41	0.89	6.69	10.12	5.00	0.45	4.12	5.88	
Overall	6.74	0.50	5.76	7.71	4.57	0.08	4.41	4.73	

^а^ -Log rank test with Mantel cox

**Fig. 6:**
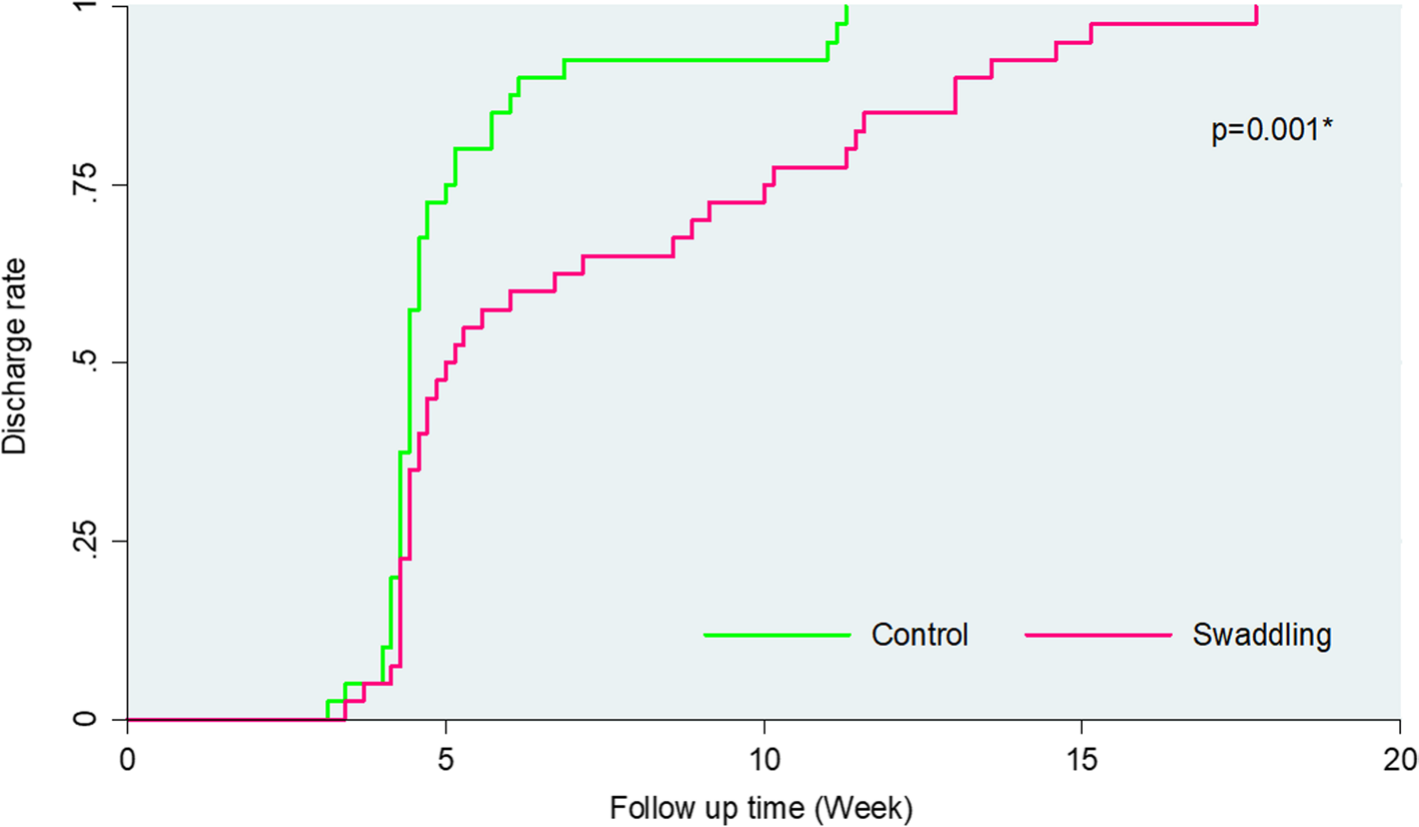
DDH cumulative discharge rate of the study groups (Kaplan Meier Curve)

Figure 6 shows the DDH cumulative survival curves of the study groups for time to discharge (Kaplan Meier Curve).

The mean duration of the swaddling was 35.3 ± 9.7 days and the mean swaddling hours per day was 20.6 ± 1.9 h. There is a correlation between the primary outcome and the swaddling frequency in hours per day (r = 0.81) and swaddling length in days (r = 0.43).

## Discussion

### Main findings

The study provides evidence that traditional swaddling is a significant contributing factor for delayed maturation of the physiologically immature Type 2a hips and development of DDH. All children were finally discharged with healthy Type 1 hips but swaddling significantly prolonged time to Type 1 hips. Furthermore, “non-Type 1” hips correlated with swaddling length per day and frequency in days.

To our knowledge, this is the first randomized controlled trial (RCT) assessing the effect of traditional swaddling on DDH. Despite the small sample size, our results show Mongolian traditional prolonged swaddling where arms and legs are extended and hips are in extension and adduction position increases the risk for DDH. Although the study was conducted in the Mongolian context, it might be a mirror of other countries where prolonged tight swaddling method is still common. In the study, quality of hip ultrasound-based diagnosis was crucial. For this purpose, a well experienced data collector/neonatologist performed the ultrasound examination and took hip sonograms of each child using the standardized Graf’s method. Also, an experienced sonographer controlled all cases at monthly basis using the standardized Graf’s method. Each hip ultrasound examination was checked and confirmed by experts using a web-based quality control platform.

### Limitations

Some limitations have to be addressed. First, the limited number of infants available for the analysis of secondary outcomes prevents us from drawing stronger statistical conclusions concerning these variables. Second, a bias might have occurred due to contamination and regarding compliance of swaddling or non-swaddling. However, we have controlled the parents’ compliance of the intervention through several arrangements: a) the allocation process was done by research assistants who are not related to the study; b) in order to standardize swaddling and non-swaddling behavior, the swaddling group was provided with usual traditional Mongolian wrapping sets and blankets while the non-swaddled group was provided warm clothing with required sizes; and c) trained research assistants made home visits to check and monitor the families to follow study protocol and to collect information on swaddling hours and frequency. Also, the sonographer and experts had no information about the initial status of the hip and study groups to prevent a bias of performers.

### Previous studies

Swaddling is still common in many countries during the first months of life due to its security and comfort, aides in calming the baby and establishing sleep patterns [7, 13]. According to a large-scale randomized controlled trial that investigated the effect of traditional swaddling on infant health and development in Mongolia, there are no harmful effects of swaddling on pneumonia rates in infants up to 7 months of age [14]; furthermore, swaddling did not have significantly affect Bayley Scales of Infant Development, especially mental and motor scale scores at around 13 months of age compared with those not swaddled [15]. Another two RCTs compared crying rates of swaddled and non-swaddled infants and swaddling was a beneficial supplementation in excessively crying infants < 8 weeks of age [16, 17]. However, the effect of swaddling on DDH was not measured in the RCTs. Therefore, the findings of those studies and our results cannot be compared.

Although, to our knowledge, no previous RCT tested the effect of swaddling on physiologically immature hips, our study results are supported by findings of a number of observational studies in different countries where swaddling was a long standing tradition. There was a 10-fold increase in DDH in Canadian Native Americans from Ontario who used prolonged cradleboard swaddling where hips are in extension and adduction position [9]; the results were supported by another study [18]. Similar concerns were noted in nomad Lapps of Sweden, where the cradleboard was believed to account for the high incidence (24.6%) of DDH [19]. A retrospective case-control study in Turkey [20] found that postnatal traditional swaddling is one of the main factor both in the etiology of DDH and in development of more severe hip dysplasia in patients with DDH; the odds ratio of DDH in swaddled children was 2.8. Another Turkey study found that the odds ratio of DDH in swaddled children was 6.1 [21]. Swaddling is also found to be responsible for the high incidence for DDH in Japan [22, 23], Saudi Arabia [24] and Hungary [25]. A retrospective study in Canada found that swaddling was more common (40% vs. 25%, OR 2.1) among infants with late presenting hip dislocation [26].

### Underlying mechanism

There might be some explanations how swaddling has played a significant contribution to the increased rates of “non-Type 1” hips and DDH in the current study.

First, the anatomical development of the hip joint. Etiology of DDH in otherwise healthy infants is multifactorial. It is generally believed that **genetic, hormonal, and/or mechanical factors** play a role [1, 2]. Studies suggest that baby hips may be more well-formed before birth and become more dysplastic around the time of birth due to postnatal influences on DDH. Lee et al. studied hip developments using ultrasonography in pre-term infants; and reported that preterm breech infants < 32 weeks’ gestational age were less likely to show abnormal ultrasound findings for DDH than those 32 to < 37 weeks of gestational age [27]. Another sequential sonographic study of hip morphology of preterm infants concluded that the normally developing hip is well-formed before birth [28]. Moreover, the study revealed that beta angles measured at birth in term infants were significantly larger than those of preterm infants indicating increased displacement or deformity of the labrum [28]. A study of fetal ultrasonography reported that prenatally, the mean alpha angles were above (at 34 weeks of gestation 61.1°, at 36 weeks 60.7° and at 38 weeks 61.2°) the level that corresponds to a mature hip joint (60°) [29]. In addition, both studies suggested that transient ligamentous laxity from maternal relaxin and abnormal mechanical pressures after birth may contribute to soft tissue deformation in the mature infant [28, 29].

Second, the initial hip type (Graf Type 2a) should also be thoroughly discussed. The Type 2a hips are comparatively prevalent, with frequencies from 10 to 45% depending on infants’ age [3, 4, 30, 31]. Although these hips have a considerable potential for spontaneous maturation, they are physiologically immature and therefore located between perfect hips and DDH and are at risk of worsening. The rate of spontaneous normalization in type 2a hips is reported to be 90–97%, whereas dysplasia persists or worsens in 3–10% of cases [31, 32]. Graf subdivides into Type 2a + and 2a- at age 6–12 weeks. In type 2a+, spontaneous maturation up to the age of 12 weeks is to be expected, while in type 2a- the assumed linear development makes this unlikely. Type 2a- is classified as DDH and treated.^11^ Two studies evaluated predictors of worsening sonographic findings; an alpha angle < 55 on the initial ultrasonography, instability, central nervous system anomalies, and unilateral Type 2a hips were found to be independent predictors of sonographic worsening [33, 34].

Third, the swaddling method is important to discuss. Not all swaddling techniques pose similar risks to the hips. The harmful effect of swaddling on DDH may be seen in cultures that swaddle with hips adducted and extended. Several studies indicate that extension of the normal infants’ hip flexion contracture may contribute to DDH including dislocation [35–37]. Also, in a rat model, straight-leg swaddling has been shown to have harmful effects on infant hips, especially in those which have been swaddled at a younger age, and those which have undergone prolonged swaddling [38, 39]. The swaddling timing and duration might indeed be another crucial issue. Swaddling is commonly used in the first 3–5 months of children’s life which is the time of significant potential for maturation time of young infants’ hip joints. This overlapping time of swaddling and maturation is potentially critical, especially for physiologically immature hips. Some epidemiological studies indicate that swaddling is a contributing factor but do not present detailed data concerning the initiation time, duration and frequency of swaddling.

## Conclusion

Mongolian traditional swaddling where arms and legs are extended and hips are in extension and adduction position increases the risk for DDH. In Mongolia, with its harsh natural environment and where nomadic herding is a major industry and way of life, traditional swaddling of newborns and infants is till common. This practice helps newborns stay warm and allows for them to be more easily carried around but parents should be made aware of the need to allow flexion and abduction of their newborn’s hips.

## Data Availability

The datasets used and/or analysed during the current study available from the corresponding author on reasonable request.

## Abbreviations

DDH: Developmental dysplasia of the hip
IQR: Interquartile range
SD: Standard deviation
CI: Confidence interval
SG: Swaddling group
NSG: Non-swaddling group
FU1: First follow-up
FU2: Second follow-up
FU3: Third follow up
FU4: Fourth follow up
OR: Odds ratio
RCT: Randomized controlled trial

## References

[1] Shipman SA, Helfand M, Moyer VA, Yawn BP (2006). Screening for developmental dysplasia of the hip: a systematic literature review for the US preventive services task force. Pediatrics.

[2] Loder RT, Skopelja EN (2011). The epidemiology and demographics of hip dysplasia. ISRN Orthop.

[3] Munkhuu B, Essig S, Renchinnyam E, Schmid R, Wilhelm C (2013). Incidence and treatment of developmental hip dysplasia in Mongolia: a prospective cohort study. PLoS One.

[4] Ulziibat M, Munkhuu B, Schmid R, Baumann T, Essig S. Implementation of a nationwide universal ultrasound screening programme for developmental dysplasia of the neonatal hip in Mongolia. J Child Orthop. 2020;14. 10.1302/1863-2548.14.200029. 10.1302/1863-2548.14.200029 PMC7453163 32874359

[5] Carter C, Wilkinson J (1964). Genetic and environmental factors in the etiology of congenital dislocation of the hip. Clin Orthop Relat Res.

[6] Skirving AP, Sims TJ, Bailey AJ (1984). Congenital dislocation of the hip: a possible inborn error of collagen metabolism. J Inherit Metab Dis.

[7] van Sleuwen BE, Engelberts AC, Boere-Boonekamp MM, Kuis W, Schulpen TWJ, L'Hoir MP (2007). Swaddling: a systematic review. Pediatrics.

[8] Mahan S, Kasser JR (2008). Does swaddling influence developmental dysplasia of the hip?. Pediatrics.

[9] Salter R (1968). Etiology, pathogenesis and possible prevention of congenital dislocation of the hip. Can Med Assoc J.

[10] National Center for Maternal and Child health (2019). Health Indicators-2019.

[11] Graf R (2006). Hip sonography: diagnosis and management of infant hip dysplasia.

[12] Essig S, Schmid R, Munkhuu B, Baumann T. Qualitätskonzept eines Ultraschall-basierten, nationalen Screeningprogramms für Hüftdysplasie in der Mongolei. Ultraschall Med-Eur J Ultrasound. 2017;38(S 01):V2-002. https://www.thieme-connect.com/products/ejournals/html/10.1055/s-0037-1606954.

[13] Oden RP, Powell C, Sims A, Weisman J, Joyner BL, Moon RY (2012). Swaddling: will it get babies onto their backs for sleep?. Clin Pediatr (Phila).

[14] Manaseki-Holland S (2005). Investigation of the effect of swaddling on lower respiratory tract infection in infants from Mongolia [PhD thesis].

[15] Manaseki-Holland S, Spier E, Bavuusuren B, Bayandorj T, Sprachman S, Marshall T (2010). Effects of traditional swaddling on development: a randomized controlled trial. Pediatrics.

[16] van Sleuwen BE, L'hoir MP, Engelberts AC, Busschers WB, Westers P, Blom MA (2006). Comparison of behavior modification with and without swaddling as interventions for excessive crying. J Pediatr.

[17] Long T (2007). Adding swaddling to behaviour modification in infant care did not reduce excessive crying in healthy infants 13 weeks of age at randomisation. Evid Based Nurs.

[18] Ghibely A (1990). Congenital dislocation of the hip in the Cree Indian population of Quebec, Canada. Acta Orthopaedica Belgica.

[19] Mellbin T (1962). The children of Swedish nomad Lapps. VII. Congenital malformations. Acta Orthop Scand.

[20] Ömeroglu H, Akceylan A, Köse N (2019). Associations between risk factors and DDH and ultrasonographic hip type: a retrospective case control study. J Child Orthop.

[21] Akman A, Korkmaz A, Aksoy MC, Yazici M, Yurdakök M, Tekinalp G (2007). Evaluation of risk factors in developmental dysplasia of the hip: results of infantile hip ultrasonography. Turk J Pediatr.

[22] Ishida K (1977). Prevention of the development of the typical dislocation of the hip. Clin Orthop Relat Res.

[23] Yamamuro T, Ishida K (1984). Recent advances in the prevention, early diagnosis, and treatment of congenital dislocation of the hip in Japan. Clin Orthop Relat Res.

[24] Abu-Nawarig M (1981). Congenital dysplasia of hip in Eastern Province of Saudi Arabia. Orthop Rev.

[25] Cz’eizel A, Szentpetery J, Kellermann M (1974). Incidence of congenital dislocation of the hip in Hungary. Br J Prev Soc Med.

[26] Mulpuri K, Schaeffer EK, Andrade J, Sankar WN, Williams N, Matheney TH (2016). What risk factors and characteristics are associated with late-presenting dislocations of the hip in infants?. Clin Orthop Relat Res.

[27] Lee J, Spinazzola RM, Kohn N, Perrin M, Milanaik RL (2016). Sonographic screening for developmental dysplasia of the hip in preterm breech infants: do current guidelines address the specific needs of premature infants?. J Perinatol.

[28] Gardiner H, Clarke NMP, Dunn PM (1990). A sonographic study of the morphology of the preterm neonatal hip. J Pediatr Orthop.

[29] Stiegler H, Hafner E, Schuchter K, Engel A, Graf R (2003). A sonographic study of prenatal hip development: from 34 weeks of gestation to 6 weeks of age. J Pediatr Orthop B.

[30] Baronciani D, Atti G, Andiloro F, Bartesaghi A, Gagliardi L, Passamonti C (1997). Screening for developmental dysplasia of the hip: from theory to practice. Collaborative group DDH project. Pediatrics.

[31] Rosendahl K, Toma P (2007). Ultrasound in the diagnosis of developmental dysplasia of the hip in newborns. The European approach. A review of methods, accuracy and clinical validity. Eur Radiol.

[32] Roovers EA, Boere-Boonekamp MM, Mostert AK, Castelein RM, Zielhuis GA, Kerkhoff TH (2005). The natural history of developmental dysplasia of the hip: sonographic findings in infants of 1-3 months of age. J Pediatr Orthop B.

[33] Bilgili F, Sağlam Y, Göksan SB, Hürmeydan ÖM, Birişik F, Demirel M (2018). Treatment of Graf type IIa hip dysplasia: a cut-off value for decision making. Balkan Med J.

[34] Kosar P, Ergun E, Gökharman FD, Turgut AT, Kosar U (2011). Follow-up Sonographic results for Graf type 2a hips association with risk factors for developmental dysplasia of the hip and instability. J Ultrasound Med.

[35] Coon V, Donato G, Houser C, Bleck EE (1975). Normal ranges of hip motion in infants six weeks, three months, and six months of age. Clin Orthop Relat Res.

[36] Salter R (1966). Role of innominate osteotomy in the treatment of congenital dislocation and subluxation of the hip in the older child. J Bone Joint Surg.

[37] Suzuki S, Yamamuro T (1993). The mechanical cause of congenital dislocation of the hip joint. Acta Orthop Scand.

[38] Ning B, Jin R, Lin W, Wang D (2018). Cellular and molecular changes to chondrocytes in an *in vitro* model of developmental dysplasia of the hip-an experimental model of DDH with swaddling position. Mol Med Rep.

[39] Wang E, Liu T, Li J, Edmonds EW, Zhao Q, Zhang L (2012). Does swaddling influence developmental dysplasia of the hip? An experimental study of the traditional straight-leg swaddling model in neonatal rats. J Bone Joint Surg.

